# Local anesthetic toxicity: acute and chronic management

**DOI:** 10.1002/ams2.265

**Published:** 2017-03-06

**Authors:** Kenichi Sekimoto, Masaru Tobe, Shigeru Saito

**Affiliations:** ^1^ Department of Anesthesiology Gunma University Graduate School of Medicine Maebashi Japan

**Keywords:** Bupivacaine, lidocaine, lipid emulsion, local anesthetic, ropivacaine

## Abstract

Local anesthetics are commonly used medicines in clinical settings. They are used for pain management during minor interventional treatments, and for postoperative care after major surgeries. Cocaine is the well‐known origin of local anesthetics, and the drug and related derivatives have long history of clinical usage for more than several centuries. Although illegal use of cocaine and its abuse are social problem in some countries, other local anesthetics are safely and effectively used in clinics and hospitals all over the world. However, still this drug category has several side‐effects and possibilities of rare but serious complications. Acute neurotoxicity and cardiac toxicity are derived from unexpected high serum concentration. Allergic reactions are observed in some cases, especially following the use of ester structure drugs. Chronic toxicity is provoked when nerve fibers are exposed to local anesthetics at a high concentration for a long duration. Adequate treatments for acute toxic reactions can secure complete recovery of patients, and careful use of drugs prevents long‐lasting neurological complications. In addition to respiratory and circulatory management, effectiveness of lipid rescue in the acute toxicity treatment has been certified in many clinical guidelines. Prevention of the use of high concentration of local anesthetics is also validated to be effective to decrease the possibility of nerve fiber damage.

## Acute Toxicities of Local Anesthetics

### Mechanisms of local anesthetic toxicity

Local anesthetics (LAs) have an ester or amine structure, and have affinity for lipid and water environments. This amphipathic chemical characteristic allows these anesthetics to cross cytoplasmic and intracellular membranes. Local anesthetics interact with charged targets, including structural proteins and signaling systems. Because of their chemical character, LAs have the possibility to produce various toxic effects in many tissues, especially heart and brain.^1^ All LAs have similar toxicity to some extent. However, the intensity of the toxicity varies among LAs, according to the chemical structure. Amine type LAs have more allergy‐inducing tendencies compared to amine type LAs.

**Figure 1:**
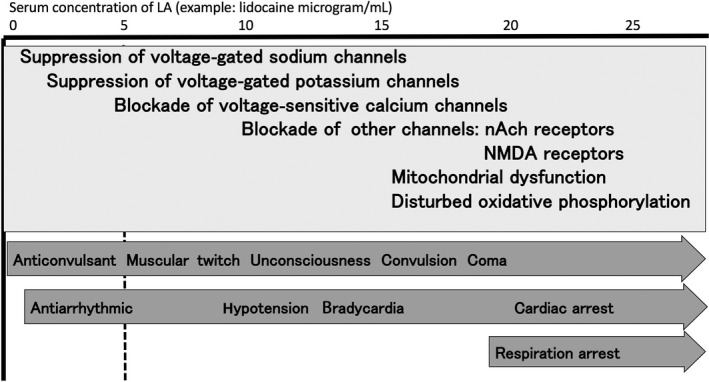
Mechanism and symptoms of acute local anesthetic toxicity. nAch, nicotinic acetylcholine; NMDA, N‐methyl‐D‐aspartate.

Although the pharmacological and toxicological acting sites of LAs are the voltage‐gated sodium channels, many alternative sites are considered to be the other targets (Fig. 1). In anesthesia clinical practice, bupivacaine is known to have potent toxicity. It interrupts both metabotropic and ionotropic signal transduction. The toxicity of bupivacaine is more apparent in tissues with high aerobic demand and low tolerance for hypoxia. Clinical symptoms of LA toxicity are seizures, cardiac arrhythmias, and hypotension (Table 1). From these clinical features, LA toxicity is expected to be derived from mitochondrial dysfunction. 2

**Table 1:** Symptoms of local anesthetic toxicity

Early neurological symptoms
Circumoral and/or tongue numbness
Metallic taste
Lightheadedness
Dizziness
Visual and auditory disturbances (difficulty focusing and tinnitus)
Disorientation
Drowsiness
Severe respiratory and cardiovascular symptoms
Hypotension
Arrhythmia
Bradycardia
Cardiac arrest
Respiratory arrest

### Epidemiology and risk factors

Several cohort studies identified rates of incidence for systemic LA toxicity associated with various clinical forms of regional anesthesia (Table 2).^3^ In most reports, rates of severe cases of toxicity (convulsive seizures with or without cardiac event) are approximately 1:10,000 for epidural analgesia and approximately 1:1000 for peripheral nerve blocks.^2^ Although these cohort data are suggestive for such risks, careful evaluation seems to be required because of many confounding factors, which make scientific interpretation difficult. For example, cardiac arrests provoked following spinal anesthesia can result from vasodilative reaction and severe hypotension. Incidence of cardiac arrests immediately after epidural anesthesia can be similarly confounded by physical reactions, such as severe sympatholysis due to cervical and/or thoracic autonomic signal blockade.

**Table 2:** Epidemiology and risk factors of local anesthetic systemic toxicity

Epidemiology
1.8: 10,000 (Otolaryngological cases; Ireland PE, Ferguson JK, Stark EJ. Laryngoscope. 1951; 61: 767–77)
79: 10,000 (Brachial plexus blocks; Brown DL *et al*. Anesth Analg 1995; 81: 321–8)
3.5: 10,000 (French anesthesiologists; Auroy Y *et al*. Anesthesiology 2002; 97: 1274–80)
Risk factors
Pre‐existing pulmonary, cardiac, and nervous vulnerabilities.
Large dose injection
Injection around vessel‐rich region
Needle or catheter placement without imaging devices
Bolus injection without aspiration test
Injection without test dosing

However, a high percentage of seizures followed by cardiac arrests are considered to be provoked by systemic toxicity of LAs.^4^ These toxic events result from direct injection of such drug into vascular space or absorption from surrounding tissues. When directly injected into vascular space, symptoms occur within a few minutes. In contrast, when absorbed from surrounding tissues, symptoms may be delayed by many minutes, or even hours.

Although most of the epidemiological studies surveyed major hospitals, many cases of LA toxicity occur in clinics or outpatient surgical centers. Of serious concern is that anesthetics, which may provoke potent toxic reactions, are often injected or provided by non‐anesthesiologists. It means that misdiagnosis or underreporting of LA‐associated complications is inevitable. Following several fatal cases of LA toxicity, provoked by lidocaine toxicity in tumescent analgesia for liposuction, notification of the risk was repeated socially, and awareness of the problem became highlighted. The most well‐known report may be the fatal cases published in 1999.^5^ Seemingly, where appropriate emergent treatment is not possible, such fatal incidents can still occur, especially in clinics where LAs are delivered without anesthesiologist's involvement. Recently, risk of LA‐containing cream was also announced, because LA applied over large areas of skin, especially when skin is wrapped by an occlusive dressing, such as cellophane, can be systemically absorbed and induce toxic levels of serum LA concentration.^6^

As the risk of LA toxicity is high among some special patients, use of a reduced dose is indicated in debilitated or acutely ill patients, small children or patients of advanced age, and patients with liver disease and/or cardiovascular disease.^4^

### Treatment and management of acute LA toxicity

When LA toxicity is suspected, the initial step to be taken is the stabilization of vital signs. If life‐threatening signs and symptoms develop during the administration of LA, immediate cessation of the injection is mandatory, and medical staff should prepare to treat the adverse reactions.^4^

#### Call for help

As treatment of LA toxicity requires a significant number of personnel, especially at the beginning of respiratory and circulatory symptoms, a number of trained staff should be called up when the first LA toxicity symptom is identified. Rapid response team members are suitable to administer treatment.

#### Secure airway

Medical staff should pay attention to impending airway problems, significant hypotension, dysrhythmias, and seizures. Once other etiologies of the patient's symptoms have been excluded, management of the symptoms specific for LA toxicity should be initiated. Adequate oxygenation should be secured first, whether by mask ventilation or by other airway management, such as intubation or laryngeal mask insertion.

#### Control seizure

There are some options for the treatment of central nervous system complications. Seizures have been treated with benzodiazepines or barbiturates successfully in many case reports. One mg/kg of i.v. propofol is also effective to stop LA‐induced seizures and convulsive muscle movement.

The American Society of Regional Anesthesia and Pain Medicine (ASRA) recommends benzodiazepines as first‐line treatment for LA‐induced seizures. These drugs have minimal potential for causing cardiac depression. When seizures persist after the use of benzodiazepines, use of small doses of muscle relaxants may be considered to ameliorate acidosis and hypoxemia induced by massive muscle contractions. When muscle relaxants are utilized, securing of the airway with intubation and ventilation are required.

When propofol or thiopental is used, it should be noted that these agents should be used at their lowest effective dose, because of their potential to worsen hypotension or cardiac depression. Also, propofol may cause bradycardia, which necessitates additional vagolytic agents. Basically, benzodiazepines are preferred to propofol in patients with signs of cardiovascular instability.

#### Stabilize circulation

**Figure 2:**
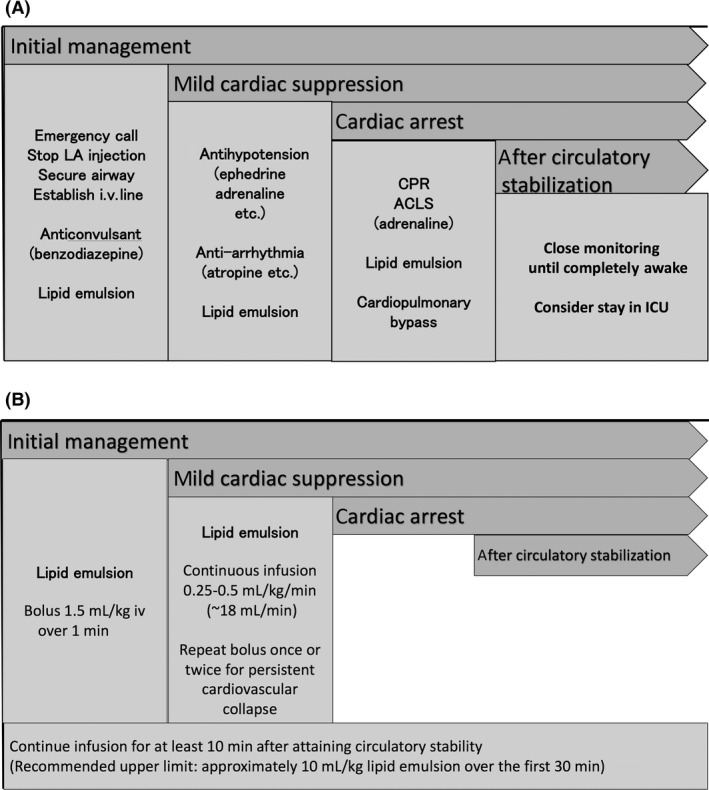
Management of acute local anesthetic (LA) toxicity. A, Sequence of symptoms and required treatments. B, Sequence of symptoms and program of lipid emulsion (20%) infusion. ACLS, advanced cardiovascular life support; CPR, cardiopulmonary resuscitation; ICU, intensive care unit.

Characteristics of arrhythmia induced by LAs are prolonged PR, QRS, and QT intervals potentiating reentry. Aberrant conduction may result in cardiac arrest (Fig. 2A). Cardiac resuscitation after such arrhythmia may be difficult and prolonged. Some LAs are highly lipophilic and require a long duration prior to redistribution. Although such cardiac toxicity is serious, properly conducted cardiopulmonary resuscitation can successfully rescue those patients. The ASRA recommends standard advanced cardiovascular life support (ACLS), with minor modifications, when cardiac arrest is provoked by LAs (Table 3). The 2015 version of the American Heart Association's ACLS guideline also described the modified regimen as “special circumstances of resuscitation”.

**Table 3:** Special considerations in cardiac life support for patients with local anesthetic‐induced cardiac arrest^a^

1) If epinephrine is used, small initial doses (10–100 μg boluses in adults) are preferable
2) Vasopressin is not recommended
3) Avoid calcium channel blockers and beta‐blockers
4) If ventricular arrhythmias develop, amiodarone is preferable
5) In patients with cardiac toxicity, avoiding the use of lidocaine and related class IB antidysrhythmic agents (e.g., mexiletine, tocainide) is crucial because they may worsen toxicity. Lidocaine has been used successfully in bupivacaine‐induced dysrhythmias, but its additive central nervous system toxicity is still a major concern.
6) In patients who do not respond to standard resuscitative measures, cardiac pacing and cardiopulmonary bypass may be introduced to improve the outcome. Cardiopulmonary bypass may serve as a bridging therapy until tissue levels of the local anesthetic have cleared.

a Adapted from ASRA guidelines.^4^

Although it is not widely accepted, combined boluses of glucose, insulin, and potassium were recommended as a successful treatment in reversing bupivacaine‐induced cardiovascular events.^7^ Another report showed a reduced central nervous system (CNS) and cardiovascular toxicity of bupivacaine when an extract of traditional Chinese herbal medicines, “shenfu”, was used on rats.^8^ These are introduced as an option in a Web‐based guidelines.

In any severe reactions, monitoring of the cardiovascular condition and circulatory support with i.v. fluids and vasopressors are indispensable. As hypoxemia and acidosis possibly potentiate the cardiovascular toxicity of LAs, early control of seizures and airway intervention to treat hypoxemia and acidosis may prevent cardiac arrest and accelerate recovery from such disastrous conditions. Sodium bicarbonate can be used to treat severe acidosis.^4^

Cardiac arrest due to LA toxicity is a rare but well‐known complication that may occur after use of a large dose. It should be noted that the patients have a favorable prognosis, if circulation can be restored by adequate and timely management. Earnest efforts in resuscitation are extremely important in LA toxicity cases. Percutaneous cardiopulmonary support has been used effectively to treat refractory cardiac arrest due to LA toxicity.^9^ It is reasonable to alert the nearest facility having cardiopulmonary bypass capability when LA toxicity signs are identified in a patient.

#### Decrease serum LA concentration by lipid emulsion

Intravenous infusion of a lipid emulsion has become part of the treatment for systemic toxicity from LAs, particularly for refractory cardiac arrest. The ASRA guidelines recommend starting lipid emulsion therapy at the first signs of systemic toxicity from LAs, after airway management (Fig. 2B).^4^ It is proposed that lipid infusion creates a lipid phase that extracts the hydrophobic molecules of LA from the aqueous plasma phase. An *in vitro* study by Mazoit *et al*. reported the high solubility of LAs in lipid emulsions and the high binding capacity of these emulsions.^10^

Weinberg *et al*. showed, in an animal experiment, that application of lipid emulsion infusion was effective in the resuscitation of bupivacaine‐induced cardiac arrest.^11^, ^12^ Rosenblatt *et al*. first reported the use of lipid infusion to resuscitate a patient from prolonged cardiac arrest provoked by an interscalene block with bupivacaine and mepivacaine.^13^ Several subsequent case reports further documented successful use of lipid emulsion in the treatment of neurologic and cardiac LA toxicity. Local anesthetics involved in these reports were ropivacaine, mepivacaine and prilocaine, and levobupivacaine.^14^, ^15^, ^16^, ^17^, ^18^, ^19^ Among such reports regarding successful application of lipid emulsion, Marwick *et al*.'s was unique, because in their case systemic toxicity recurred 40 min after the successful lipid rescue. This report stressed the importance of the availability of a sufficient quantity of lipid emulsion where large volumes of LA solution are used for regional anesthesia.^20^

Several recommendations were proposed for the lipid emulsion therapy as follows: (i) lipid emulsion therapy is carried out with a 20% solution; (ii) administer a bolus of 1.5 mL/kg over 1 min; (iii) following infusion may be at a rate of 0.25 mL/kg/min for 20 min, 30–60 min, or until hemodynamic stability is restored; and (iv) when adequate resuscitation cannot be attained, bolus doses may be repeated up to two times, possibly at 5‐min intervals, until stable rhythm is restored. Alternatively, the infusion rate may be increased (e.g., to 0.5 mL/kg/min for 10 min). The recommended upper limit of lipid emulsion is approximately 10 mL/kg over the first 30 min.

Weinberg *et al*. have shown that lipid emulsion therapy provides superior hemodynamic and metabolic recovery from bupivacaine‐induced cardiac arrest than either epinephrine or vasopressin.^21^, ^22^ However, Mayr *et al*. reported that vasopressin combined with epinephrine resulted in better short‐term survival rates than lipid emulsion in a porcine model of bupivacaine toxicity.^23^ Harvey *et al*. showed that ACLS with lipid emulsion resulted in lower rates of spontaneous circulation compared with ACLS alone in a rabbit asphyxial model.^24^ Also, several adverse events have been reported after a rapid lipid emulsion infusion (Table 4).^25^

**Table 4:** Adverse events reported after a rapid lipid emulsion infusion

Acute kidney injury
Cardiac arrest
Ventilation–perfusion mismatch
Acute lung injury
Venous thromboembolism
Hypersensitivity
Fat embolism
Fat overload syndrome
Pancreatitis
Extracorporeal circulation machine circuit obstruction
Allergic reaction
Increased susceptibility to infection

#### Treatments for allergy and methemoglobinemia

It does not often occur, however, LAs do have the possibility of provoking an allergic or hematologic reaction. Allergic reactions can be treated with diphenhydramine or, for more serious reactions, epinephrine or corticosteroids. Methemoglobinemia should initially be treated symptomatically. In severe cases, methylene blue and hyperbaric oxygen may be utilized.^4^

#### Cares for prevention

The prevention of LA toxicity should be considered primarily. Although all adverse effects cannot be anticipated, many complications can be evaded or minimized by strict adherence to the guidelines of LA dose.^4^, ^26^, ^27^ Identification of high‐risk patients and implementation of appropriate LA application techniques, and adequate vital sign monitoring, are effective to avoid disastrous scenarios of LA toxicity. Fundamental precautions, shown in Tables 2and 5, may help to avoid complications related to LA use, especially in emergency department patients. A careful injection method is known to prevent toxic reactions. Large‐volume injections should be undertaken slowly and incrementally. Intermittent aspiration and observing for blood in the syringe are also mandatory. Injecting LA in such a careful manner reduces the chances of a large‐volume intravascular injection.

**Table 5:** Tips to avoid local anesthetic toxicity

1) Consider obtaining informed consent in patients with a history of anesthetic reactions
2) Document the amount and type of anesthetic used during the procedure
3) Obtain an adequate history and physical examination to identify risk factors and allergies
4) Do not use class IB antidysrhythmics for seizures or dysrhythmias due to cocaine toxicity
5) Consider neurologic signs or symptoms as a manifestation of anesthetic toxicity
6) Admit patients with serious symptoms
7) Know the toxic dose of the local anesthetic
8) Use the lowest concentration and volume of local anesthetic that is still effective
9) Add epinephrine at a ratio of 1:200,000 to slow vascular uptake
10) Describe the early symptoms of local anesthetic overdose to patients
11) Instruct patients to inform the physician if they experience any uneasiness
12) Be sure that patients understand the effects of local anesthetics and emphasize that they should tell the physician if symptoms occur

Maintaining verbal contact with the patient during the procedure is helpful to notify LA toxicity at an early stage. This helps to detect subtle symptoms, such as dysarthria, as well as changes in mental status and consciousness. As benzodiazepines increase the threshold for seizure, benzodiazepine premedication may result in direct cardiovascular collapse, skipping signs of CNS toxicity.

## Chronic Neurotoxicity

### Chronic neurotoxicity of LAs: effects on neurites and growing neural edges

Local anesthetics were suggested to have a potential for neurotoxicity in both clinical reports and laboratory experiments.^2^ Such phenomenon has been widely notified following a series of case reports that described long‐lasting neurological disorder provoked by intrathecal lidocaine infusion.^28^ However, precise morphological changes induced by the direct application of LAs to neurons have not yet been fully understood.

In nerves to which lidocaine was applied, inflammation together with reactive fibrosis has been observed around nerve fibers near the administration site. In the histopathologic assessments, the infiltrating cells were mainly macrophages, giant cells of foreign body type, lymphocytes, and plasma cells.^29^ Although most experimental nerve damage and clinical neurological disorders can be recovered within several months, there are multiple case reports describing cases with long‐lasting, severe neurological disorders following LA injection.^28^ Laboratory studies proposed multiple mechanism of the neurological damage induced by LAs (Table 6). 30

**Table 6:** Proposed mechanism of chronic toxicity of local anesthetics on nerve fibers

Effects on nerve cell body: membrane lysis, apoptosis
Effects on nerve fibers: electrophysiological effects, delay in axonal transport
Effects on edge of growing nerve fibers: growth cone collapse

### Effects of LAs on crude growing neuritis

Observation of living nerve reactions to LA application is helpful to understand the toxicity of LAs on nerve fibers. However, despite the fact that LAs are sometimes applied to sites where peripheral nerves may be regenerating after injury, the effects of LAs on growing or regenerating neurons have been rarely studied. Growing or regenerating neurons might be susceptible to the toxic effects of LAs.

To examine the effects of LAs on growing neurons, we adopted a growth cone collapse assay, which is a quantitative measurement of neuronal morphological changes induced by externally applied substances.^31^ The growth cone is the leading edge of an extending neurite, and it has crucial roles in pathfinding and cytoskeletal organization during neuronal development.^32^ Then, by morphologically observing growth cones and growing neurites, actions of externally applied substances to growing nervous tissues can be clearly identified.

We examined the effect of several LAs on three different types of growing neurons isolated from chick embryos: sympathetic, peripheral sensory (nerves from dorsal root ganglion), and retinal (a part of central neuron).^33^ Tetracaine induced growth cone collapse and neurite destruction. Three neuronal tissues showed significantly different dose response. The growth cone collapsing effect was partially reversible.

When intracellular Ca(2+) concentration was measured by Fura‐2‐acetoxymethyl ester after exposure to tetracaine, tetracaine simultaneously induced collapse and Ca(2+) increase at growth cones.^34^ The Ca(2+) hot spot was expanded into the neurite from the periphery towards the cell body. When tetracaine was applied to growth cones in Ca(2+)‐free media, the increase was minor.

This neurotoxic potential of LAs is similar among different LAs.^35^ We observed that all of the LAs examined, lidocaine, bupivacaine, mepivacaine, and ropivacaine, produced growth cone collapse and neurite degeneration. However, they showed significant differences in the dose response. The half maximal inhibitory concentration (IC_50_) values were approximately 10exp(−2.8) mol/L for lidocaine, 10exp(−2.6) mol/L for bupivacaine, 10exp(−1.6) mol/L for mepivacaine, and 10exp(−2.5) mol/L for ropivacaine at 15 min of exposure. Some reversibility was observed after replacement of the media. At 20 h after washout, bupivacaine and ropivacaine showed insignificant percentage growth cone collapse in comparison to their control values, whereas those for lidocaine and mepivacaine were significantly higher than the control values.

### Supporting actions of neurotrophic factors

In several studies, the role of some neurotrophic factors (NTFs) in supporting developing neurons exposed to the deleterious effects of these drugs was examined, and the beneficial effects of some trophic factors have been reported.^36^ For example, after 60 min of exposure to lidocaine, the culture media were replaced to wash out the lidocaine. When any of the three NTFs, brain‐derived neurotrophic factor, glial‐derived neurotrophic factor, or neurotrophin‐3, was added to the replacement media at a minimum concentration of 10 ng/mL, significantly high reversibility of the lidocaine‐induced growth cone collapse was observed, especially at 48 h after washout. At that time point, there was no significant difference between the values of growth cone collapse percentage in the cells that were exposed to lidocaine and supported by the NTFs after the washout, and the control cells (not exposed to lidocaine). Similarly, when any of the NTFs were used after the washout of bupivacaine or mepivacaine, the collapsing activity was significantly attenuated, and growth cone collapse values showed no statistically significant differences in comparison with the pre‐exposure values obtained prior to the application of LAs.^37^

### Neural effects of LAs at a lower concentration

In a more recent study, the effects of prolonged exposure to LAs at a lower concentration were studied.^38^ Neurite growth was delayed significantly when an LA was applied at a relatively low concentration. Filopodia of growth cones retracted, and their number was significantly decreased after the application of LA. The quantity of actin in cell bodies increased, contrary to the effect on neurites and growth cones, suggesting that axonal transport of actin is disrupted.

Local anesthetics delay or interrupt the neurite extension when applied to growing or regenerating neurons. This effect can be deleterious for normal establishment and maintenance of nervous tissues. However, where abnormal sproutings provoke neurological disorder, this inhibitory action of LAs might be beneficial to maintain the normal neuronal circuits.^39^ This toxicological knowledge seems to be crucial, when physicians consider the future clinical applications of LAs.

### Prevention and treatment of chronic neurological disorder provoked by LAs

**Figure 3:**
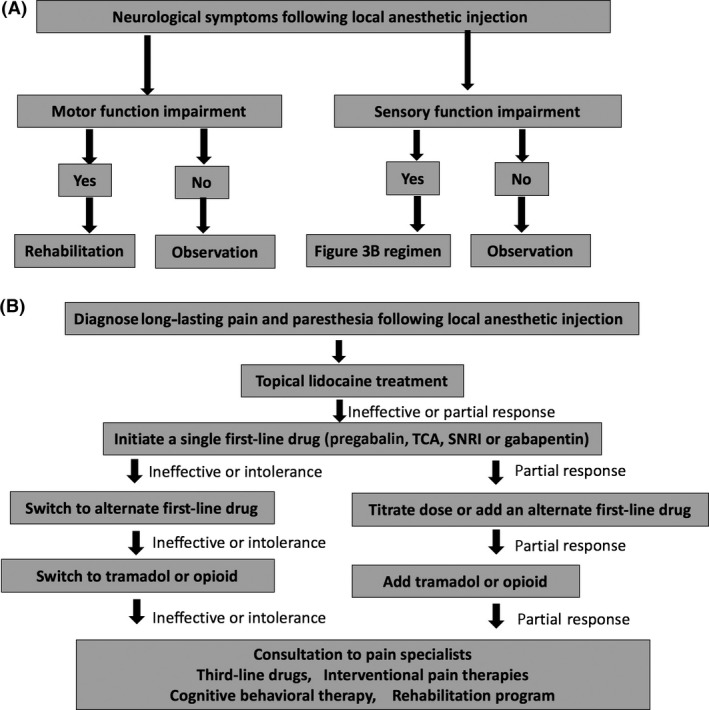
Management of chronic local anesthetic (LA) toxicity. A, Basic treatment algorithm for chronic LA toxicity. B, Treatment algorithm for chronic sensory neurological symptoms derived from LA toxicity. SNRI,: serotonin–norepinephrine reuptake inhibitor; TCA, tricyclic antidepressant.

To prevent chronic toxicity of LAs on nerve fibers, physicians should apply LA carefully, especially when LA is injected near nerve fibers (Fig. 3).^2^, ^29^ The main points are: cautious use at a low concentration (lidocaine should not be used at high concentration, especially adjacent of nerve fibers), and intrathecal continuous infusion should be carefully utilized.

Therapies for LA‐induced chronic nerve damage are mostly identical with therapies for neuropathic pain.^40^ Usually, non‐steroidal anti‐inflammatory drugs are ineffective, and tricyclic antidepressants, anticonvulsants, serotonin noradrenalin reuptake inhibitors, and opioids may be used, depending on the patient's symptoms and severity. Spinal cord electrical stimulation and cognitive behavioral therapy are also possible options usually adapted by pain therapy specialists. As many studies are ongoing both for mechanism clarification and for therapeutic development, we recommend further reading of updated reports regarding chronic neurotoxicity of LAs.^41^

### Perspectives

Local anesthetic toxicity is now well known among clinical staff, especially those working in acute medicine. Treatment guidelines for acute toxicity have been increasingly established and revised periodically by some specialist societies. Public announcements through the Internet are also actively promoted. Many descriptions in this review article are based on such sources.^4^, ^6^, ^26^, ^27^, ^42^ Understanding and prevention strategies against chronic neurological damage by LAs are also prevailing among anesthesiologists and neurologists. However, treatment for chronic neurological disorder provoked by LAs is not necessarily successful and still under investigation. This therapeutic incompleteness is common for other types of neuropathic pain and disorders. Further basic and clinical studies seem to be indispensable to establish more effective and reliable treatment guidelines.

## Conflict of Interest

None declared.
